# Weight Gain and *De Novo* Metabolic Disorders after Liver Transplantation

**DOI:** 10.3390/nu11123015

**Published:** 2019-12-10

**Authors:** Barbara Lattanzi, Daria D’Ambrosio, Daniele Tavano, Demis Pitoni, Gianluca Mennini, Stefano Ginanni Corradini, Massimo Rossi, Manuela Merli

**Affiliations:** 1 Department of Translational and Precision medicine, Sapienza University of Rome, Italy Viale dell’Università 37, 00185 Roma, Italy; lattanzi.b@gmail.com (B.L.); daria.dambrosio@uniroma1.it (D.D.); daniele.tavano@uniroma1.it (D.T.); pitonidemis@gmail.com (D.P.); stefano.corradini@uniroma1.it (S.G.C.); 2 Department of General Surgery “Paride Stefanini”, Liver Transplantation Unit, “Sapienza” University, 00185 Rome, Italy; gianluca.mennini@uniroma1.it (G.M.); massimo.rossi@uniroma1.it (M.R.)

**Keywords:** liver transplantation (LT), body mass index (BMI), metabolic disorders (MDs), non-alcoholic steatohepatitis (NASH)

## Abstract

The development of nutritional and metabolic abnormalities represents an important burden in patients after liver transplantation (LT). Our study aimed at evaluating the incidence, time of onset, and risk factors for nutritional and metabolic abnormalities in patients after LT. The study was a single-center retrospective study. Consecutive patients undergoing elective LT from 2000 to 2016 were enrolled. The presence of at least two among arterial hypertension (AH), diabetes mellitus (DM), dyslipidemia, and obesity (BMI ≥ 30 Kg/m^2^) was utilized to define patients with the metabolic disorder (MD). Three hundred and fifteen patients were enrolled; the median age was 56 years (68% males). Non-alcoholic steatohepatitis (NASH) was the origin of liver disease in 10% of patients. During follow-up, 39% of patients developed AH, 18% DM, and 17% dyslipidemia. Metabolic disorders were observed in 32% of patients. The NASH etiology (OR: 6.2; CI 95% 0.5–3; *p* = 0.003) and a longer follow-up (OR: 1.2; CI 95% 0.004–0.02; *p* = 0.002) were associated with de novo MD. In conclusion, nutritional and metabolic disorders are a frequent complication after LT, being present in up to one-third of patients. The NASH etiology and a longer distance from LT are associated with de novo MD after LT.

## 1. Introduction

The development of nutritional and metabolic abnormalities represents an important burden in patients after liver transplantation (LT) [1]. Immunosuppressive therapy, sedentary lifestyle, increase in appetite, and changes in eating habits are all contributing factors.

After, LT patients may become overweight and even morbidly obese [1,2]. More recently, non-alcoholic steatohepatitis (NASH) is increasing as a cause of liver disease which may lead to liver cirrhosis and end-stage liver disease. Non-alcoholic steatohepatitis is the most rapidly rising indication for LT in the United States, and it is projected to become the most common indication in future years [3,4]. Patients with a diagnosis of NASH are frequently overweight or obese [5,6], and nutritional and metabolic disorders have been found to persist or rapidly recur after LT [7,8,9].

The chronic use of immunosuppressants, particularly corticosteroids and calcineurin inhibitors, may also contribute to worsen metabolic disorders and to weight gain [2]. Indeed, corticosteroids promote insulin resistance through downregulation of insulin production, upregulation of hepatic gluconeogenesis, and a decrease in glucose utilization in peripheral tissues [10,11]. Calcineurin inhibitors (CNIs), tacrolimus, and cyclosporine are known to increase vasoconstriction and cause sodium-dependent volume expansion leading to arterial hypertension [12]. Calcineurin inhibitors have also been associated with decreased insulin sensitivity and reduced insulin release [13]. Moreover, both CNIs and mammalian target of rapamycin (mTOR) inhibitors (sirolimus and everolimus) increase serum lipid levels, the latter affecting the levels more severely [14].

An increase in body weight, arterial hypertension, and serum glucose levels at 6 months have been reported as predictors of metabolic syndrome after LT [15]. Furthermore, metabolic disorders may also favor cardiovascular diseases, increasing cardiovascular mortality in the long term after LT [16]. 

In this setting, patients with metabolic disorder after LT constitute a high-risk group in which therapeutic interventions should be optimized [9,17]. 

Our study aimed at evaluating the incidence, time of onset, and risk factors for nutritional disorders and de novo metabolic abnormalities in patients after LT.

## 2. Materials and Methods

The study was a single-center retrospective study. The medical records of all patients undergoing elective LT at the University Hospital Policlinico Umberto I in Rome from 2000 to 2016 were reviewed. Exclusion criteria were age < 18 years, re-transplant, combined kidney–liver transplant, and a follow-up ≤ 3 months. All patients were followed from the date of transplantation until death, loss to follow-up or end of study (15 June 2019). Age, gender, body mass index, liver disease etiology, immunosuppressive treatment (at discharge and maintenance), and time of corticosteroid therapy were collected in each patient. Body mass index (BMI), diagnosis of diabetes mellitus (DM), arterial hypertension (AH), and dyslipidemia were derived from clinical records before LT and at 1, 3, 5, and 10 years after LT. Body weight before liver transplant was always corrected for water retention. Metabolic disorders were defined as de novo when they appeared after LT. The presence of at least two among AH, DM, dyslipidemia, and BMI ≥ 30 Kg/m^2^ during follow-up was arbitrarily utilized to define patients as those with metabolic disorder (MD).

All patients were followed at the outpatient clinic of the Transplant Centre of our University Hospital. The first-line standard immunosuppressive therapy was triple therapy with steroids, CNIs, and mycophenolate mofetil (MMF); steroids were generally continued for 3–6 months and MMF for 1 year. In the case of autoimmune etiology, the steroid therapy was maintained chronically at low doses; in the case of kidney dysfunction, MMF was continued or reintroduced together with CNIs to maintain the lowest dose of CNIs. Everolimus (EVR) was also alternatively utilized to discontinue CNIs completely in patients with kidney dysfunction. 

Results are expressed as the mean ± standard deviation, median, and range or as percentage as indicated. For the comparison among groups, the Pearson chi square test or the Fischer exact test were used for categorical variables. For continuous variables, the Mann–Whitney test was applied. A multivariate logistic regression analysis was utilized to identify factors independently associated with the development of metabolic disorders after LT. Only variables with a *p*-value < 0.05 at univariate analysis were included.

The paired “*t*” test was utilized for the analysis of repeated variables. Values of *p* < 0.05 were considered statistically significant.

## 3. Results

### 3.1. Study Population 

**Figure 1:**
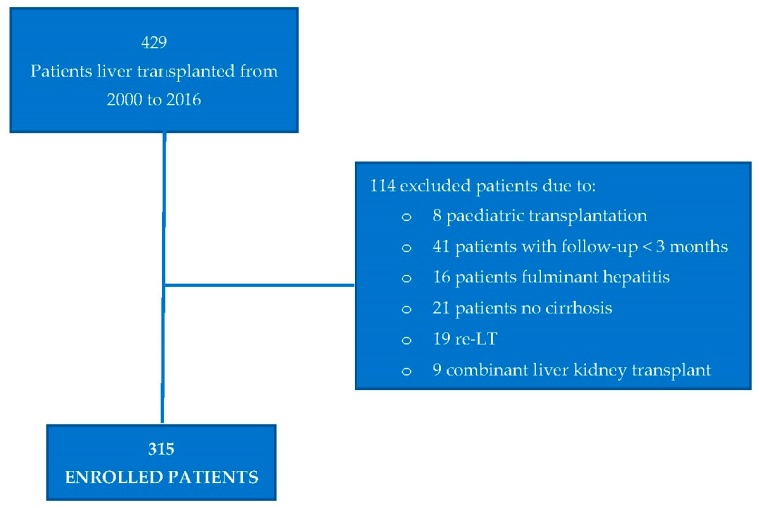
Flowchart of the study.

A total of 315 patients submitted to liver transplantation were enrolled in the study (Figure 1), the median age was 56 years (range 18–68), 68% were males, and the most frequent origin of LT was the hepatitis C virus, followed by alcohol abuse. Thirty-one patients (10%) were transplanted for NASH. Before LT, 18% of patients presented AH, 28% DM, and 12% dyslipidemia; a BMI ≥ 30 was present in 14% of patients. Seventeen percent of patients had at least two metabolic disorders. Demographic and clinical characteristics of patients at transplant are shown in Table 1. 

### 3.2. Weight Gain After LT

**Figure 2:**
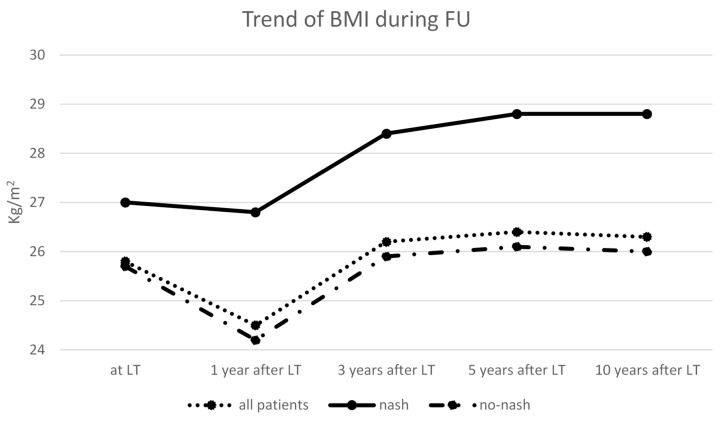
BMI modifications in 315 subjects followed after liver transplantation. All patients (dotted line), patients with previous NASH (continue line), patients with no-NASH (dashed–dotted line). * <0.05 compared to BMI at LT.

Figure 2 shows the trend of BMI after LT. Mean BMI was slightly but significantly decreased 1 year after LT (25.8 ± 4 versus 24.5 ± 5 Kg/m^2^, *p* = 0.03; basal versus 1 year, respectively). Later on, BMI tended to gradually increase and became significantly higher versus pre-transplant levels in the third year (24.5 ± 5 versus 26.2 ± 4 Kg/m^2^, *p* = 0.04 basal versus 3 years, respectively), reaching a plateau at 5 and 10 years of follow-up. Patients transplanted for NASH showed a different pattern: BMI was unchanged at 1 year in comparison to pre-LT condition (27 ± 3 versus 26.8 ± 4 Kg/m^2^; *p* = 0.6, basal versus 1 year, respectively), while it increased progressively at 3 (28.4 ± 5 Kg/m^2^, *p* = 0.05 versus basal) and 5 years (28.8 ± 4 Kg/m^2^, *p* = 0.05 versus basal) (Figure 2).

### 3.3. Metabolic Disorders After LT

**Figure 3:**
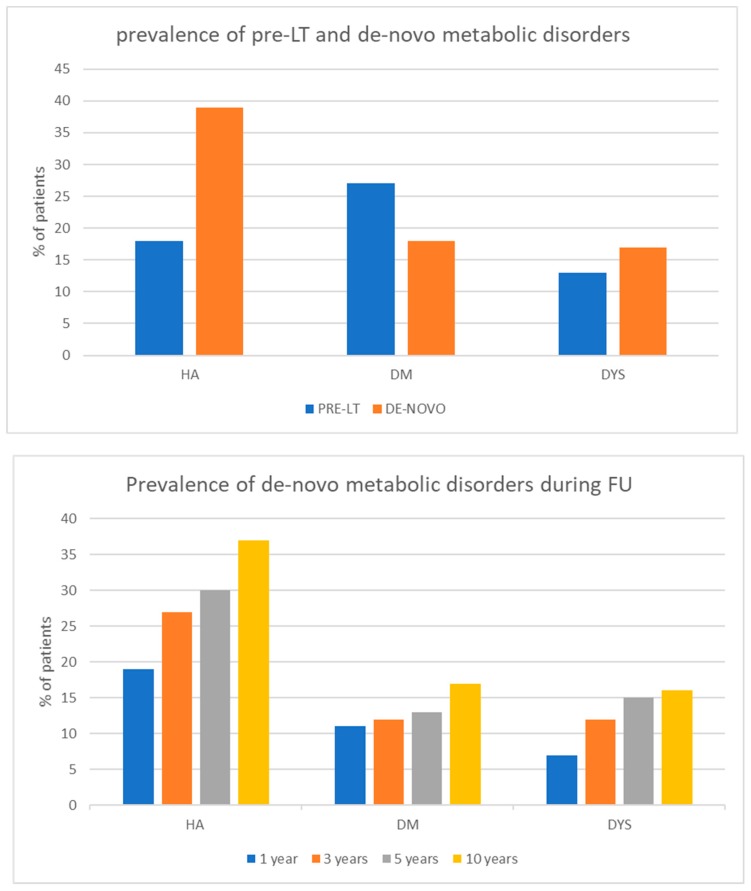
Prevalence and time of onset of de novo metabolic disorders after LT.

During a median follow-up of 75.5 months (range 3–220 months), 122 patients (39%) developed de novo AH after LT. Fifty percent of de novo AH was diagnosed within the first year after LT. De novo DM was diagnosed in 57 patients (18%) during the follow-up. Fifty-nine percent of patients developed this diagnosis within the first year after LT. De novo dyslipidemia was diagnosed in 53 patients (17%), 39% of them developed this disorder within the first year after surgery (Figure 3). 

At least two de novo metabolic disorders were reported in 32% of overall patients; of this, 42% were diagnosed during the first year, 67% within the third year after LT, and 82% within the 5th year. Patients transplanted for NASH showed the highest rate of de novo DM, HA, and MD (Table 2).

### 3.4. Risk Factors Associated with De Novo Metabolic Disorders

The univariate analysis identified NASH, time of follow-up after LT, a higher BMI before and at 1 year from LT as risk factors for de novo MD. The use of tacrolimus seemed to be protective for the development of MD (Table 3). The multivariate analysis selected NASH etiology (OR: 6.2; CI 95% 0.5–3; *p* = 0.003) and a longer FU (OR: 1.2; CI 95% 0.004–0.02; *p* = 0.002) as the only independent risk factors associated with the development of de novo MD.

## 4. Discussion

Metabolic disorders and weight gain are important burdens in patients after LT. These are due to the fact of multiple reasons such as a sedentary lifestyle, increased appetite, and modifications in eating habits [1]. The chronic use of immunosuppressants may also play a role in the development of these conditions [2].

Weight gain after liver transplantation is welcome in patients transplanted with severe nutritional depletion; however, a rapid weight gain might be harmful. In our population, mean BMI at the time of LT was only slightly above normal values; however, these patients were likely overhydrated during end-stage liver disease; therefore, BMI was possibly overestimated. The majority of our patients were therefore normal or undernourished at transplantation. Mean BMI, in our series, showed a trend to decrease the first year after surgery and tended to recover later on. This result is at variance with studies showing a rapid and severe weight gain after liver transplantation [9,18,19]. This difference might depend on a lower prevalence of patients with NASH origin in our study (only 10%). Indeed, in the previously cited studies, the percentage of patients transplanted for NASH was much greater. In the 31 NASH patients in our series, we observed that BMI progressively increased from the first year after surgery was significantly higher at 3 and 5 years. A higher percentage of patients transplanted for NASH is therefore likely to modify the pattern of weight changes after LT throughout a more frequent development of post-transplant obesity. In this regard, bariatric surgery has been proposed before or concomitant to LT in patients with morbid obesity [20].

In our study, we found a high rate of de novo metabolic disorders (AH, DM, dyslipidemia) after LT. Our finding is in keeping with what has been previously reported [1,19,21]; however, most of the former studies did not focus on de novo metabolic disorders as in the present investigation. 

The majority of de novo disorders developed, in our study, in the first year after LT. In the subgroup of patients transplanted for NASH, de novo AH, DM, and MD occurred even at a higher rate. 

It has been suggested that immunosuppressive strategies (the kind of immunosuppressant, the dosages, and the period length of steroid assumption) may play a role in the rate of development of de novo AH, DM, dyslipidemia, and MD, after LT [22]. In our study, we failed to find a correlation with MD and the different immunosuppressive regimens adopted. Tacrolimus appeared to have a protective role in the onset of de novo MD at univariate analysis but was not found to be an independent predictor at multivariate analysis. The retrospective nature of our study has certainly limited the possibility to follow all the dose modifications of the immunosuppressive therapy which is frequently tailored in the individual patients according to nephrotoxicity, rejection risk or clinical events.

The onset of de novo metabolic disorders after LT, not surprisingly, was associated with NASH as the origin of the previous liver disease and increased during the time. A longer distance from transplantation is likely to identify those patients with the longest time of exposure to immunosuppressants and those with older age. Previous studies reported a similar association among older age and obesity, AH, DM, and metabolic syndrome after LT [23,24,25]. 

Our study has some limitations, being a monocentric and retrospective study. The retrospective analysis of our database prevented the possibility to diagnose a metabolic syndrome according to the proposed cut-offs as HDL cholesterol and the abdominal circumference were not reported in the database. For this reason, we utilized the term “metabolic disorders” to represent the development of relevant metabolic disorders (at least two among AH, DM, dyslipidemia, and obesity) in our patients. 

## 5. Conclusions

In conclusion, de novo HA, DM, dyslipidemia, and obesity are frequent in liver transplant patients, mostly in those with NASH etiology. These latter patients also experience a progressive weight gain which contributes to metabolic disorders. Interventions to minimize the risk of developing weight gain and metabolic disorders after LT need to be planned. Giving particular care to those patients at higher risk.

## Figures and Tables

**Table 1:** Demographic and clinical characteristics of the 315 patients at the time of liver transplantation (LT).

Variable	Patients (*n* = 315)
Age at LT (years)	56 (18–68)
MELD score	15 (6–40)
BMI (Kg/m^2^) *	25.3 (17–38)
BMI ≥ 25, *n* (%) *	138 (44%)
BMI ≥ 30, *n* (%) *	44 (14%)
Male gender, *n* (%)	209 (68%)
Etiology, *n* (%)	
HCV	118 (37%)
HBV	54 (17%)
NASH	31 (10%)
Alcohol	67 (21%)
Other	45 (15%)
HCC, *n* (%)	141 (45%)
AH pre-LT, *n* (%)	58 (18%)
DM pre-LT, *n* (%)	86 (28%)
Dyslipidemia pre-LT, *n* (%)	33 (12%)
At least two metabolic disorders (MD), *n* (%)	54 (17%)
Immunosuppressive treatment at discharge, *n* (%)	
Triple therapy with TAC (steroids + MMF + TAC)	259 (82%)
Triple therapy with EVR (steroids + MMF + EVR)	7 (2%)
Dual therapy with TAC (steroids + TAC)	45 (15%)
Other	4 (1%)
Duration of steroids treatment (months)	6.5 (0–125)
Follow-up (months)	75.5 (3–220)

Continuous variables expressed as median (range). Abbreviations: LT—liver transplantation; HCC—hepatocellular carcinoma; BMI—body mass index; MELD—model for end-stage liver disease; HCV—hepatitis C virus; HBV—hepatitis B virus; NASH—non-alcoholic steatohepatitis; AH—arterial hypertension; DM—diabetes mellitus; MMF—mycophenolate mofetil; TAC—tacrolimus; EVR—everolimus. * BMI refers to body weight corrected for water retention.

**Table 2:** Incidence of metabolic disorders before and after liver transplantation in patients transplanted or not for non-alcoholic steatohepatitis.

Variable	Transplanted for NASH 31 Patients	Transplanted for Other Etiology 294 Patients	*p*-Value
**De novo DM**	**10 (32%)**	**47 (16%)**	**0.03**
**De novo AH**	**20 (64%)**	**102 (34%)**	**0.007**
De novo Dyslipidemia	7 (23%)	46 (14%)	0.5
**De novo MD**	**17 (55%)**	**85 (28%)**	**0.003**
Pre-LT DM	8 (26%)	78 (26%)	0.89
Pre-LT AH	5 (16%)	53 (18%)	0.7
**Pre-LT Dyslipidemia**	**16 (51%)**	**17 (6%)**	**<0.0001**
**Pre-LT MD**	**14 (45%)**	**36 (12%)**	**<0.0001**

Abbreviations: NASH—non-alcoholic steatohepatitis; DM—diabetes mellitus; AH—arterial hypertension; LT—liver transplantation; MD—metabolic disorder. The statistically significant data are in bold.

**Table 3:** Univariate analysis for the development of metabolic disorder status after liver transplant.

Variable	De Novo MD 102	Non-De Novo MD 213	*p*-Value
Male gender, n (%)	73 (72%)	136 (64%)	0.2
Age (years)	54 ± 8	54 ± 11	0.9
**Etiology NASH, n (%)**	**26 (25%)**	**5 (3%)**	**<0.0001**
**BMI pre-LT (kg/m^2^)**	**26.9 ± 3**	**25.6 ± 4**	**0.01**
**BMI 1 year after LT (kg/m^2^)**	**26.4 ± 5**	**24 ± 4**	**0.02**
MELD	14 ± 5	16 ± 7	0.1
Immunosuppressive drugs at discharge			
Steroids	98 (96%)	200 (94%)	0.2
MMF	87 (85%)	176 (83%)	0.6
AZA	14 (14%)	21 (10%)	0.3
**Tacrolimus**	**77 (76%)**	**191 (90%)**	**0.01**
Everolimus	20 (2%)	42 (2%)	0.9
Cyclosporine	24 (24%)	27 (13%)	0.09
Immunosuppressive treatment at discharge			
Triple therapy (steroids + MMF + TAC/EVR)	92 (90%)	179 (84%)	0.3
Dual therapy (steroids + TAC)	11 (10%)	34 (16%)	
Immunosuppressive drugs as maintenance			
**Steroids**	**4 (4%)**	**35 (17%)**	**0.02**
MMF	27 (26%)	53 (25%)	0.9
AZA	8 (8%)	6 (3%)	0.3
**Tacrolimus**	**65 (64%)**	**176 (83%)**	**0.007**
Everolimus	19 (19%)	234 (11%)	0.2
Cyclosporine	20 (20%)	27 (13%)	0.2
Immunosuppressive maintenance treatment			
**Triple Therapy (steroids + MMF + TAC/EVR)**	0	19 (9%)	**0.03**
Dual Therapy (steroids + TAC)	32 (32%)	61 (29%)	0.6
Monotherapy (TAC/EVR)	67 (66%)	142 (67%)	0.7
Time of steroid therapy	10 ± 9	12 ± 16	0.4
**FU months**	**114 ± 48**	**86 ± 56**	**0.001**

Abbreviations: LT—liver transplantation; BMI—body mass index; MELD—model for end-stage liver disease; NASH—non-alcoholic steatohepatitis; MMF—mycophenolate mofetil; TAC—tacrolimus; EVR—everolimus; MDs—metabolic disorders. The statistically significant data are in bold.
